# No difference in revision rate in patella‐friendly total knee arthroplasty with or without patella resurfacing at 5 years' follow‐up. A database analysis

**DOI:** 10.1002/jeo2.70448

**Published:** 2025-10-17

**Authors:** Etienne Massardier, Ophélie Manchec, Emilie Bérard, Sébastien Lustig, Etienne Cavaignac

**Affiliations:** ^1^ Department of Lower Limb Orthopaedic and Traumatology Surgery Edouard Herriot University Hospital, Hospices Civils de Lyon Lyon France; ^2^ Department of Orthopaedic and Traumatology Surgery Pierre‐Paul Riquet University Hospital Toulouse Cedex 9 France; ^3^ Department of Epidemiology and Public Health Toulouse University Hospital, CERPOP, INSERM‐University of Toulouse UPS Toulouse France; ^4^ Department of Orthopaedics Surgery and Sports Medicine FIFA Medical Center of Excellence, Croix‐Rousse Hospital, Hospices Civils de Lyon, Lyon North University Hospital Lyon France

**Keywords:** anterior knee pain syndrome, knee arthroplasty, revision surgery

## Abstract

**Purpose:**

Numerous studies have been published on the optimal management of the patella during total knee arthroplasty (TKA), but controversy remains. The aim of this study was to compare survivorship and functional outcomes with or without patella resurfacing, using a single TKA implant.

**Methods:**

This retrospective database analysis included 6078 TKA of the same cruciate‐sacrificing, mobile‐bearing, ‘patella‐friendly’ model implanted between March 2002 and December 2018 for primary osteoarthritis. TKAs implanted for inflammatory arthritis or bone tumour were excluded. The implant's survivorship was estimated at 5 years' follow‐up using the Kaplan–Meier method. Functional assessments consisted of the KSS score and knee flexion.

**Results:**

The 1641 patients who had at least 5 years' follow‐up were separated into two groups: patella resurfacing (PR) (*n* = 656) and no patella resurfacing (NPR) (*n* = 985). Mean age was 69.8 years [25–96] and mean follow‐up time was 98.4 months [60–247]. There was no difference in survivorship without reoperation between the two groups (98.4% [95% confidence interval [CI] 97.8–99] vs. 98.4% [95% CI 97.6–99.1; *p* = 0.95), nor in the mean KSS score (181.7 [95% CI 158.35–205.0] vs. 179.9 [95% CI 156.5–203.2]; *p* = 0.51). Survival free of patella‐related revisions was 99.3% [95% CI 98.9–99.7] in the NPR group versus 99.7% [99.3–100.0] in the PR group (*p* = 0.02). Mean knee flexion was significantly less in the NPR group than in the PR group (116.1° [95% CI 104.5–127.8] vs. 118.8° [95% CI 106.7–131.0]; *p* = 0.03).

**Conclusion:**

Systematic patella resurfacing when doing TKA using the SCORE™ implant does not reduce the overall revision rate and does not improve functional scores. When only the revisions for patella‐related complications are considered, the revision rate is lower in the NPR group. Knee flexion is better in patients who underwent patellar resurfacing, but this difference did not reach the clinically relevant threshold.

**Level of Evidence:**

Level III, retrospective cohort study.

**Abbreviations**
AKP: anterior knee pain
ASA: American Society of Anaesthesiologists
BMI: body mass index
CI: confidence interval
KSS: Knee Society System
MD: missing data
mFA: mechanical femoral angle
mTA: mechanical tibial angle
NPR: no patella resurfacing
PR: patella resurfacing
PROMS: patient‐reported outcome measures
TKA: total knee arthroplasty

## INTRODUCTION

Total knee arthroplasty (TKA) is a reliable, commonly performed surgical procedure that has a low complication rate and rapid recovery [5, 24, 34]. Although TKA is a very successful procedure, patellar complications can lead to postoperative pain and discomfort. The incidence of patellar complications after TKA is 4%–40% [4, 6, 11, 27, 32, 40]. The most common patellar complications after TKA are instability, anterior knee pain and misalignment [4, 6, 11, 27, 32, 40]. They are frequently the reason for early reoperation [10], including secondary resurfacing or medial patellofemoral ligament reconstruction [22].

Whether or not to systematically resurface the patella remains controversial despite multiple clinical trials and meta‐analyses on this topic. Prior studies have failed to demonstrate the superiority of either strategy. Randomised trials [10, 20] lack of statistic power and have failed to demonstrate any difference between these strategies. Although the Knee Arthroplasty Trial [3, 28] was one of the largest controlled trials, there was no significant difference in the clinical outcomes. Some biases may have compromised the results, such as the use of different implants, different techniques and inclusion of unicompartmental arthroplasty in the study design [10, 21]. Large scale database analyses and meta‐analyses [7, 8, 11, 14, 29, 31, 36, 37] have the same drawbacks and include implants of different designs. We wanted to reduce the biases associated with these two types of studies by conducting a large database analysis featuring a single ‘patella‐friendly’ TKA implant [18, 21, 23].

Our primary aim was to assess the difference in the all‐cause revision rate 5 years after TKA surgery between patients who undergo patella resurfacing (PR) and patients who do not (NPR). Our secondary aim was to compare the reoperation rate, knee range of motion and functional scores in both groups. We hypothesised that were no differences in outcomes whether or not the patella is resurfaced when using a patella‐friendly TKA implant.

## MATERIALS AND METHODS

### Surgery

**Figure 1:**
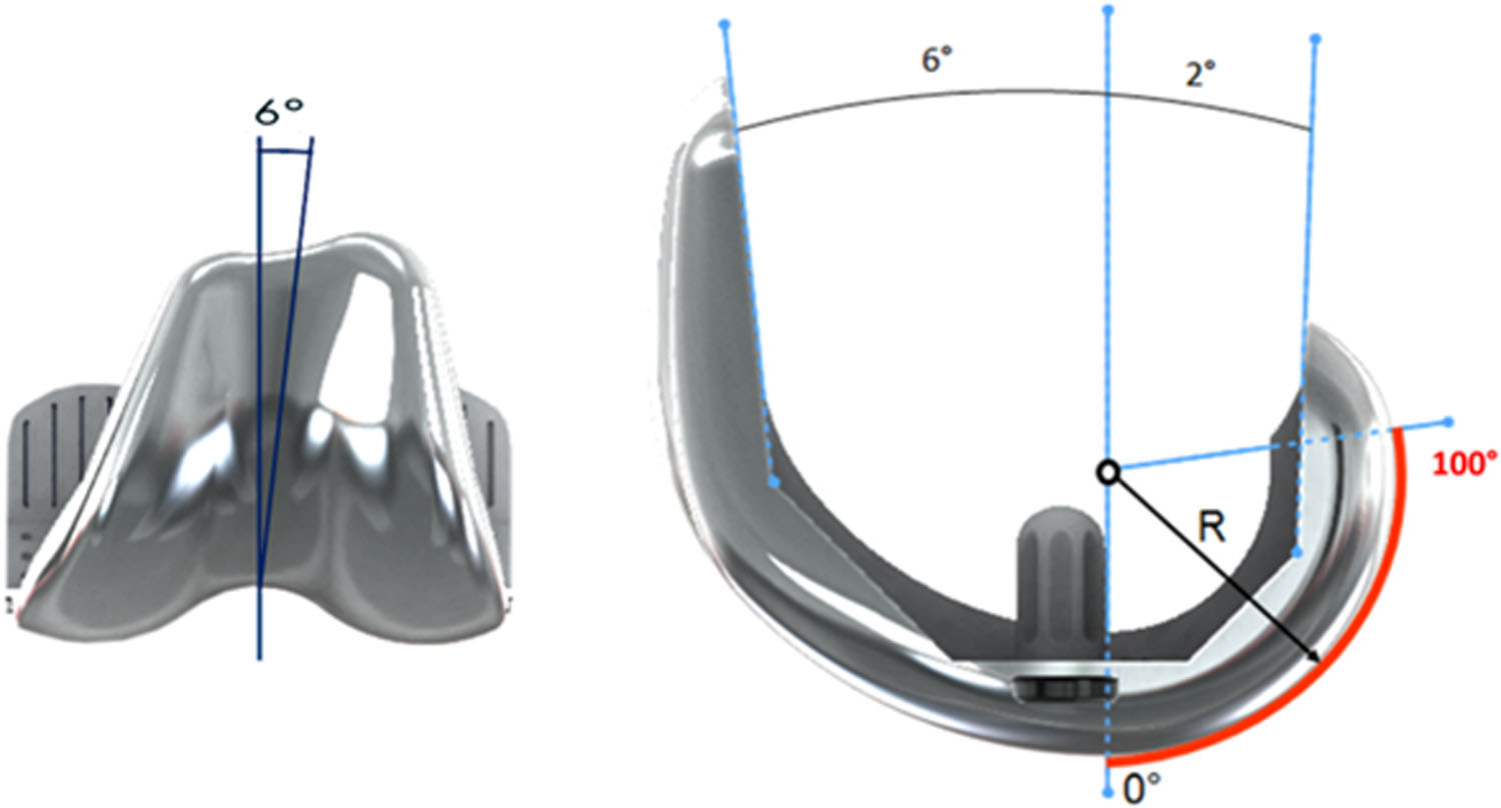
The SCORE (Amplitude, Valence, France) femoral component. PR, patella resurfacing; TKA, total knee arthroplasty.

**Figure 2:**
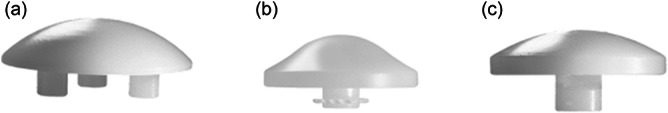
The SCORE patella buttons. (a) Onset cemented. (b) Inset non‐cemented. (c) Inset cemented.

A total of 6194 primary TKA implanted between March 2002 and December 2018 in 16 different centres were extracted from a prospective arthroplasty database maintained by an orthopaedic implant manufacturer (Amplitude, Valence, France). All the patients received a SCORE™ primary TKA implant (Amplitude) (Figure 1). This PCL‐sacrificing, rotating bearing, posterior‐stabilised implant is said to be ‘patella‐friendly’. The patellar groove has a 6° orientation and a constant radius of curvature. Its consistent depth ensures constant pressure over the patella's track. This TKA implant uses a posterior‐referenced cutting guide for femoral rotation and anterior offset. Three patellar components were available (Figure 2): 8 mm thickness inset cemented (23, 26 and 29 mm), 8.5 mm thickness inset cementless (23, 26 and 29 mm) and 7 mm thickness onset cemented (30, 33, 36 and 39 mm). Seven femoral component sizes were available, with each having a constant trochlear groove depth.

The surgeries were performed through different approaches (antero‐medial, antero‐lateral), with different fixation techniques (all cemented, cementless, hybrid), with or without navigation and with different patella implants (cemented our uncemented, inlay or onlay). Information about the surgical procedures can be found in Table 1. The Amplivision (Amplitude) navigation system was used for the procedures with navigation. We do not have information about the coronal alignment goal; however, the mechanical alignment strategy was widely used during the study period.

**Table 1:** Information about the surgical procedure.

	No resurfacing *N* = 3415 (56.2%)	Patella resurfacing *N* = 2663 (43.8%)	*p* value
Surgical approach			<0.01
Medial parapatellar	2769 (81.1%)	2361 (88.7%)	
Lateral parapatellar	314 (9.2%)	125 (4.7%)	
Missing data	332 (9.7%)	177 (6.6%)	
Tourniquet used			<0.01
Yes	220 (6.4%)	53 (2.0%)	
No	2322 (68.0%)	1784 (67.0%)	
Missing data	873 (25.6%)	826 (31.0%)	
Cement used			<0.01
None	2912 (85.3%)	1637 (61.5%)	
Tibia	228 (6.7%)	207 (7.8%)	
Femur	125 (3.7%)	252 (9.4%)	
Tibia + Femur	150 (4.4%)	567 (21.3%)	
Missing data	0 (0%)	0 (0%)	
Surgical navigation used			<0.01
Yes	2337 (68.4%)	1265 (47.5%)	
No	1078 (31.6%)	1398 (52.5%)	
Missing data	0 (0%)	0 (0%)	
Type of patella implant			‐
Inset cementless	N/A	547 (20.5%)	
Inset cemented		608 (22.8%)	
Onset cemented		1508 (56.6%)	
Missing data		0 (0%)	

### Data collection

Clinical and radiological data were collected prospectively by the lead surgeon every year during the first 5 years of follow‐up, and then at the surgeon's discretion. Radiological assessment included a minimum of anteroposterior, lateral, skyline and long‐leg anteroposterior standing views. Data were collected in a prospective database (CliniRecord, Amplitude). The make‐up of the database (No. 1355265) followed the national data privacy board (CNIL) recommendations, and the database was authorised and registered on the public platform ‘Health Data Hub’ under No. F20210913151920.

The primary outcome was survival of the implant, with revision of any of the components (implantation, removal or change) as the endpoint. Reoperation was defined as a new surgery on the same knee, for any reason, whether or not the implants were changed. Thus, every revision was also tabulated as a reoperation. Reoperation for patella resurfacing only was defined as a revision. Patients lost to follow‐up after 5 years were censored on the date of the last appointment and were not considered as an event. Secondary outcomes were the maximum knee flexion in degrees and the Knee Society System (KSS) functional score [17] at a minimum follow‐up of 5 years. Complications and reasons for reoperation were compiled.

### Statistical analysis

For the primary endpoint, a sample size of 2663 patients with patella resurfacing and 3415 patients without patella resurfacing was needed to achieve a power greater than 95% to detect equivalence with a margin of ±1.5%, with a reference group proportion expected to 98% and an actual difference fixed to 0. The significance level was 0.05.

Before any statistical analyses, verification of missing or aberrant or inconsistent data was conducted. After corrections, the database was locked. The analysis was performed on the locked database. All reported *p‐*values were two‐sided, and the significance threshold was <0.05.

Statistical analyses were performed using XLSTAT 2023.1.1 (Lumivero, Denver, CO, USA).

Patient characteristics were described by the mean value with 95% confidence intervals (CI) for continuous variables, and the number of non‐missing observations with frequency (%) for categorical variables. A Shapiro–Wilk test was used to assess the normality of the distribution for the continuous variables.

For the survival endpoints, Kaplan–Meier survival curves were generated together with 95% CI and compared first using the log‐rank test. A Cox model adjusted for age, sex, body mass index (BMI), use of navigation, use of cement and type of surgical approach was then used to determine how these factors affected survivorship.

Student's *t*‐test was used to compare the distribution of continuous secondary endpoints when the data were distributed normally (i.e., HKA at follow‐up). Mann–Whitney's test was used to compare the distribution of continuous secondary endpoints when the data distribution departed from normality or when the homoscedasticity assumption was not met (i.e., all other continuous endpoints).

## RESULTS

### Demographics

**Figure 3:**
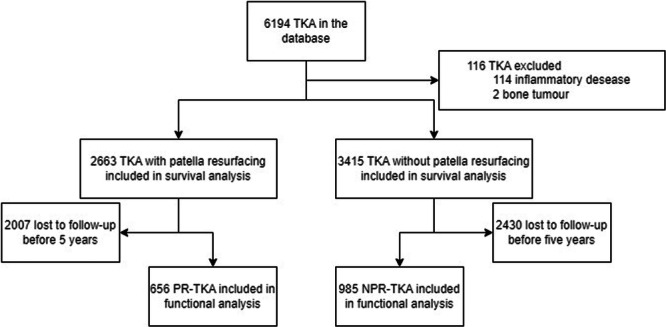
Flowchart for the study. NPR‐TKA, TKA without patella resurfacing; PR‐TKA, TKA with patella resurfacing; TKA, total knee arthroplasty.

On the 6194 TKA procedures included in the database, 116 were excluded because they were performed for inflammatory arthritis or bone tumour. Thus, 6078 patients were included in the survival analysis; however, 4437 did not have 5 years of follow‐up and were excluded from the functional analysis (Figure 3). The mean follow‐up for the whole cohort was 40.5 months (1–247). Characteristics of both groups can be found in Table 2.

**Table 2:** Patient characteristics.

	No resurfacing *N* = 3415 (56.2%)	Patella resurfacing *N* = 2663 (43.8%)	*p* value
Age in years, mean (95% CI)	70.9 (70.6–71.2)	72.2 (71.9–72.5)	<0.01
Gender *n* (%)			<0.01
Female	1764 (51.7%)	1417 (53.2%)	
Male	1651 (48.3%)	1246 (46.8%)	
Side *n* (%)			0.81
Right	1872 (54.8%)	1810 (68.0%)	
Left	1444 (42.3%)	799 (30.0%)	
Missing data	99 (2.9%)	54 (2.0%)	
BMI in kg/m² mean (95% CI)	27.9 (27.6–28.2)	28.1 (27.7–28.4)	0.52
ASA score [1]			<0.01
1	260 (7.6%)	314 (11.8%)	
2	1769 (51.8%)	1606 (60.3%)	
3	855 (25.0%)	533 (20.0%)	
4	16 (0.5%)	8 (0.3%)	
Missing data	515 (15.1%)	202 (7.6%)	
HKA in ° mean (95% CI)	176.6 (176.4–176.8)	177.3 (177.1–177.6)	<0.01
mFA in ° mean (95% CI)	91.3 (91.1–91.4)	92.6 (92.4–92.7)	<0.01
mTA in ° mean (95% CI)	87.0 (86.9–87.3)	87.6 (87.4–87.8)	<0.01
Flexion in ° mean (95% CI)	113.3 (112.8–113.8)	110.2 (109.7–110.7)	<0.01
KSS score [17]	96.1 (95.1–97.0)	89.8 (88.7–90.8)	<0.01

Abbreviations: ASA, American Society of Anaesthesiologists; BMI, body mass index; HKA, hip knee angle; KSS, Knee Society Score; mFA, mechanical femoral angle; mTA, mechanical tibial angle.

### Survivorship

**Figure 4:**
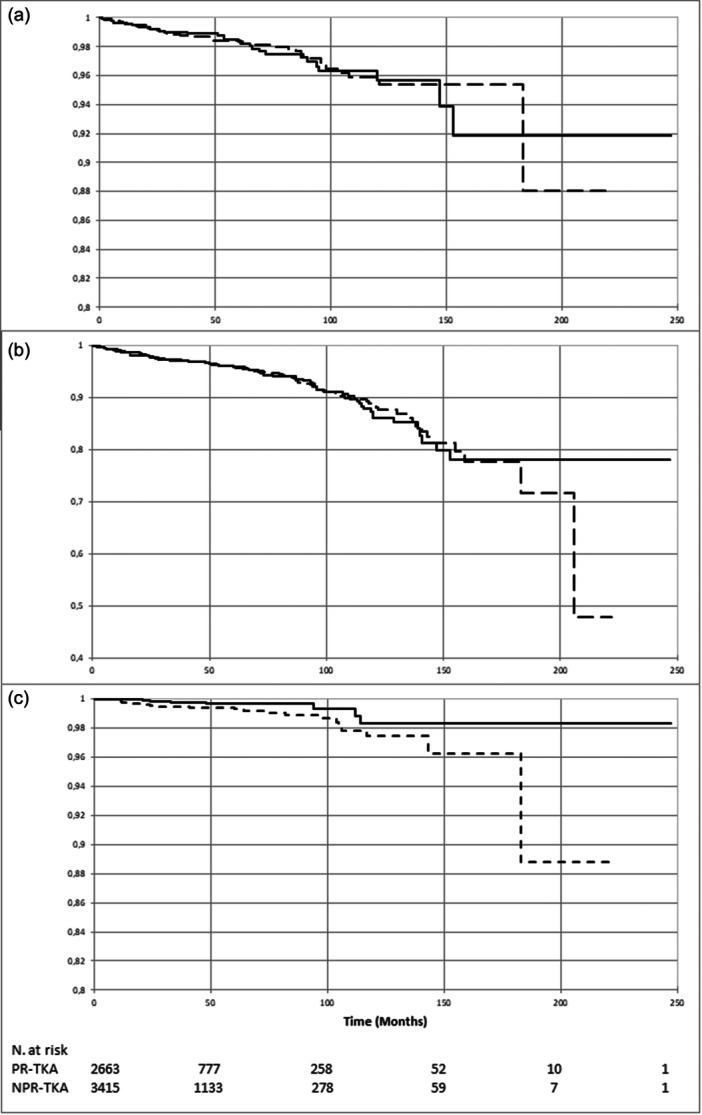
Kaplan–Meier estimates of the cumulative survival risk. Solid line: patella resurfacing. Dotted line: no resurfacing. (a) Revision of any of the implants as the endpoint (*p* = 0.95). (b) Any reoperation as the endpoint (*p* = 0.83). (c) Reoperation for patella‐related complication (*p* = 0.02). NPR‐TKA, TKA without patella resurfacing; PR‐TKA, TKA with patella resurfacing; TKA, total knee arthroplasty

The cumulative survival rate with implant revision as the endpoint at 5 years was 98.4% [95% CI 97.8–99] in the NPR group and 98.4% [95% CI 97.6–99.1] in the PR group (*p* = 0.95). When reoperation was the endpoint, whether or not the implant was removed, the cumulative survival rate was 96.0% (95% CI 95.1–96.9) versus 96.0% (95% CI 94.9–97.1) (*p* = 0.83). With revision for patella‐related complications as the endpoint, the cumulative survival rate was 99.3% (95% CI 98.9–99.7) in the NPR group versus 99.7% (99.3–100.0) in the PR group (*p* = 0.02) (Figure 4). The time to patella‐related revision was 43.6 months (range 4–125) on average in the NPR group and 32.3 months (range 2–94) in the PR group. In the Cox model, neither age, BMI, sex, surgical approach, use of navigation or use of cement had a significant impact on survival (Table 3). The main complications leading to reintervention were stiffness, septic arthritis and periprosthetic fractures. They are listed in Table 4.

**Table 3:** Factors included in the Cox analysis and resulting hazard ratios.

	HR [95% CI]	*p*‐Value
Age	0.96 [0.89–1.03]	0.32
Body mass index	0.99 [0.978–1.02]	0.61
Gender		
Male	1	
Female	0.83 [0.51–1.35]	0.45
Approach		
Medial parapatellar	1	
Lateral parapatellar	1.46 [0.68–3.13]	0.829
Navigation used		
Yes	1	
No	0.78 [0.46–1.30]	0.33
Cement used		
Tibia + Femur	1	
Tibia	1.01 [0.34–2.98]	0.99
Femur	0.39 [0.08–1.86]	0.24
None	0.94 [0.38–1.82]	0.65

**Table 4:** Number (frequency) and types of complications leading to reoperation.

Complication	No resurfacing (*n* = 3415)	Patella resurfacing (*n* = 2663)	*p* value
Aseptic loosening (except patella)	8 (0.2%)	7 (0.3%)	0.82
Pain	5 (0.1%)	4 (0.2%)	0.97
Periprosthetic fracture	26 (0.8%)	22 (0.8%)	0.40
Knee dislocation	0 (0%)	2 (0.1%)	0.11
Stiffness	46 (1.3%)	27 (1.0%)	0.23
Patella‐related	23 (0.7%)	7 (0.3%)	0.02
Septic arthritis	26 (0.8%)	28 (1.1%)	0.23

### Functional outcomes

The 1641 patients who had more than 5 years of follow‐up were included in the analysis of functional outcomes. The mean follow‐up time was 99.0 month [range 60–222] in the PR group and 92.9 months [range 60–247] in the NPR group. The functional score did not differ between the two groups: mean KSS Score at 5 years was 181.7 [95% CI 158.35–205.0] in the PR group versus 179.9 [95% CI 156.5–203.2] in the NPR group (*p* = 0.51). Mean flexion at 5 years of follow‐up was 116.1° [95% CI 104.5–127.8] in the NPR group versus 118.8° [95% CI 106.7–131.0] in the PR group (*p* = 0.03).

## DISCUSSION

The most important finding of this study was that patella resurfacing during TKA did not affect the revision‐free survival rate. Despite a difference in the rate of patella‐related complications between the two groups associated with an alpha risk of 2%, the overlapping confidence intervals do not allow us to draw conclusions about a statistically significant difference. Furthermore, it seems to be counterbalanced by other types of complications and does not affect the overall revision rate.

Our findings are consistent with previously published studies. In a randomised trial involving patients scheduled for a same‐day bilateral TKA with a patella resurfacing randomised to one side, Koh and al. [20] found no difference on PROMS, anterior knee pain or revision rate whether the patella was resurfaced or not. Similar results were found by Ha et al. [16]. Coory et al. [8] reported a statistically higher rate of revision in the Australian registry when no patella resurfacing was performed, with a difference of 1.0%–2.3%. No difference was found in the patella‐related revision rate. Fu et al. [13] reported a reduction of the risk of reoperation by 4% when the patella was resurfaced. Teel et al. [38] reported a reduction of the k of reoperation for anterior knee pain, but not for other patella‐linked complications. Thus, case‐based management of the patella seems to be a valid approach. Franck et al. [12] highlighted four parameters linked to secondary resurfacing and anterior knee pain that can be used to guide personalised management of the patella. This is consistent with other studies [29, 41] which showed that routinely resurfacing the patella was not effective in nonarthritic patellofemoral joint.

In our database analysis, the PR and NPR groups had similar functional outcomes. While there was a significant difference in mean knee flexion, it was less than the 3.8°–8.8° minimal clinically important difference [9, 15, 26, 35]. Various studies have reported better clinical or radiological outcomes when the patella is resurfaced [16, 31, 39]. But specific patella‐linked parameters and PROMS were rarely evaluated in these studies. In a meta‐analysis of ten trials comparing PR‐TKA and NPR‐TKA, Fu et al. [13] found that only seven reported anterior knee pain. And despite a statistically significant finding, Teel et al. [38] concluded that the difference in clinical scores was not clinically relevant, like in our study. Maniar et al. [25] reported a 99.7% survival rate at 15 years when taking patella revision as an endpoint and found good functional results in a retrospective study of 2520 TKA procedures with systematic resurfacing; however, 4.8% of patients had postoperative anterior knee pain despite patella resurfacing.

Benazzo et al. [2] mentioned the role of patellar morphology on the outcomes when the patella is not resurfaced and recommend resurfacing the patella if there is a mismatch between the patellar morphology and the flange. Despite good biomechanical reasons for using ‘patella‐friendly’ implants, a recent meta‐analysis by Simpson et al. [36] did not find better results in terms of anterior knee pain and functional scores when compared with other implants. Nevertheless, the reoperation rate was significantly higher in NPR group for ‘non‐patella‐friendly’ implants but was not significantly different in the ‘patella‐friendly’ implants. Kuo et al. [21] reported that even these specifically designed implants fail to restore the anatomical patellofemoral offset and frequently lead to understuffing of the patellar groove, which can explain the lack of evidence in favour of “patella friendly” implants when the patella is not resurfaced. This understuffing could lead to extensor mechanism weakening and patella maltracking. New concepts in knee arthroplasty may help to manage the patellofemoral compartment. For example, kinematic alignment has been shown to better restore the native patellar tracking and anatomic parameters after TKA [19, 30, 33].

Our study was novel in that it included only one TKA implant design, reducing the risk of biases resulting from differences in trochlear flange design. The database analysis provided high statistical power. During the study period (2002–2018), none of the procedures were robotic‐assisted, and all of them used the mechanical alignment strategy, as kinematic alignment was not used in France during that period. This made the study population more homogeneous.

Our study has limitations. Since it was a database analysis, a high number of patients were lost to follow‐up. Nevertheless, the large sample size contributed to high statistical power. And while the functional score used was not specific to the patellofemoral joint, it is the most widely used score for determining TKA outcomes. Because of the non‐randomised design, there were differences between the PR and NPR groups in the patient characteristics including age, gender and surgical procedure (approach, implant fixation and use of navigation); however, these parameters were included in the Cox proportional hazards model and did not alter our findings. Future large database or registry studies on patella‐related complications should focus on a specific implant model to reduce the variability related to implant design.

## CONCLUSION

Systematic patella resurfacing when doing TKA using the SCORE™ patella‐friendly implant does not reduce the overall revision rate and does not improve functional scores. While there were more revisions for patella‐related complications in the NPR group, it did not impact the overall reoperation rate.

## AUTHOR CONTRIBUTIONS

**Etienne Massardier**: Statistical analysis; article writing. **Ophélie Manchec**: Data collection; manuscript review. **Emilie Bérard**: Statistical analysis; manuscript review. **Sébastien Lustig** and **Etienne Cavaignac**: Study design; final approval of the manuscript.

## CONFLICT OF INTEREST STATEMENT

Sébastien Lustig received royalties from Stryker and Smith & Nephew and institutional support from Amplitude. Etienne Cavaignac is consultant for Arthrex, Biobank and Amplitude. The other authors declare no conflicts of interest.

## ETHICS STATEMENT

Data were collected in a prospective database (CliniRecord, Amplitude). The make‐up of the database (No. 1355265) followed the national data privacy board (CNIL) recommendations, and the database was authorised and registered on the public platform “Health Data Hub” under No. F20210913151920.

## Data Availability

The data that support the findings of this study are available from Amplitude, Valence, France. Restrictions apply to the availability of these data, which were used under license for this study.
